# Static and Dynamic Differences in Fixation Stability between a Spacer Plate and a Small Stature Plate Fixator Used for High Tibial Osteotomies: A Biomechanical Bone Composite Study

**DOI:** 10.1155/2013/387620

**Published:** 2013-09-23

**Authors:** Stefan Maas, Arnaud Diffo Kaze, Klaus Dueck, Dietrich Pape

**Affiliations:** ^1^Faculty of Science, Technology and Communication, University of Luxembourg, 6 rue R. Coudenhove-Kalergi, 1359 Luxembourg, Luxembourg; ^2^Department of Orthopedic Surgery, Centre Hospitalier de Luxembourg, 78 rue d'Eich, 1460 Luxembourg, Luxembourg; ^3^Sports Medicine Research Laboratory, Public Research Centre for Health, Centre Médical de la Fondation Norbert Metz, 76 rue d'Eich, 1460 Luxembourg, Luxembourg

## Abstract

*Background*. The objective of the present study was to compare mechanical strength and stability of the newly designed spacer plate with the gold standard plate for the treatment of medial knee joint osteoarthritis. *Materials and Methods*. Ten fourth-generation tibial bone composites underwent a medial open-wedge high tibial osteotomy (HTO) according to standard techniques, using five TomoFix plates and five Contour Lock plates. Static compression load to failure and load-controlled cyclical fatigue failure tests were performed. Forces and horizontal displacements were measured; plastic deformations and dynamic stiffness were determined. *Results and Discussion*. In all samples, rotation of the tibial head and fracture of the opposite cortex were observed. Behaviors of the specimens under static loading were comparable between groups. Cyclic testing revealed lateral significant higher stiffness until failure for the Contour Lock compared to the TomoFix plate. No visible implant failure was observed in any group. *Conclusion*. Considering the static analysis, both plates offered sufficient stability under physiologic loads of up to 3000 N. The Contour Lock plate-fixated specimens showed a higher stability during the cyclic testing, supposedly due to the wider distance between the fixation screws.

## 1. Introduction

High tibial closing wedge osteotomy is a well-established treatment for medial femorotibial osteoarthritis in the varus knee. The goal of osteotomy is a slight valgus overcorrection to shift the load to the intact lateral compartment of the femorotibial joint. Although clinical results after high tibial osteotomy often are encouraging, some factors are associated with a poor long-term outcome such as imprecise osteotomy or loss of the primary correction angle.

A fractured lateral cortex of the proximal tibia may lead to loss of valgus correction before bony fusion is achieved and may even necessitate reoperation. In larger correction (<8°), an opposite cortex fracture is frequent and inevitable. In these cases, maintenance of correction depends solely on the primary implant stability prior to complete healing. For the time being, the long and rigid T-shaped internal fixator (TomoFix, Synthes Gmbh, Oberdorf, Switzerland) seems to be the gold standard since it provides sufficient primary stability until solid bone healing is achieved. In addition, the TomoFix has a narrow proximal design which allows for a biplanar osteotomy, thus enlarging the surface for rapid contact healing [1, 2].

However, newly designed angle-stable implants (Contour Lock HTO, Arthrex, Karlsfeld, Germany) have been introduced recently. They have a shorter but wider proximal design with an anatomically shaped body.

**Figure 1:**
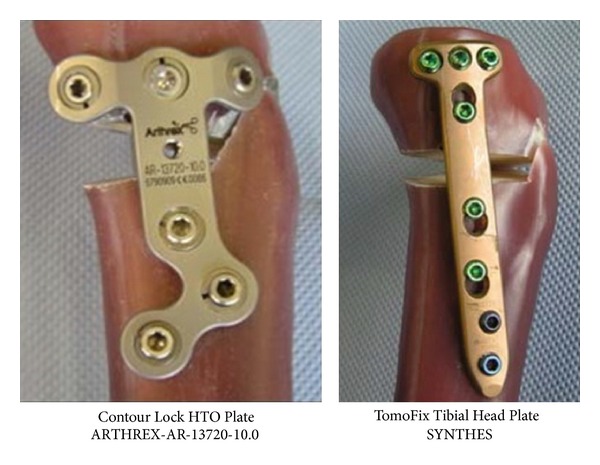
The plates are precontoured to fit the medial tibial metaphysis. The TomoFix is longer and the Contour Lock is wider.

So far, there are no biomechanical studies that quantify and compare the stabilizing effect of the Contour Lock with TomoFix plates. The present study compares the mechanical strength and stability of bone-implant constructs with the Contour Lock HTO and the TomoFix plate under static and cyclic axial loading to failure tests (Figure 1).

## 2. Materials and Methods

### 2.1. Specimen Preparation

Ten large-size fourth-generation composite analogue tibia bone models (Sawbones, Pacific Research Laboratories, Inc., Vashon, WA) were used in this study. It has been reported that these composite bones have mechanical properties similar to those of human bones [3–5]. The use of artificial composite bones provided the advantage of minimizing biomechanical variability between the specimens. The Contour Lock HTO plate (Arthrex, Medizinische Instrumente GmbH, Karlsfeld, Germany) is a short plate of about 71 mm length with locking screws and a spacer block. And the second tested implant was the TomoFix small stature plate (Synthes Gmbh, Oberdorf, Switzerland) which is a long rigid fixator plate of 112 mm length and locking screws. Both plates are precontoured to fit the medial tibial metaphysis. The TomoFix small stature plate has been designed to carry patients' weights up to 65 kg, whereas the contour Lock plate is not restricted. There is no financial relation with the commercial parties mentioned directly or indirectly in this paper.

**Figure 2:**
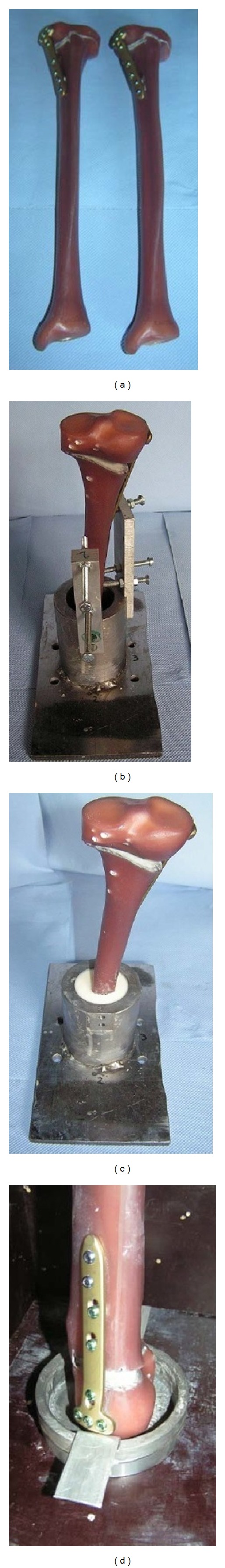
Different steps of a specimen preparation. (a) The composite bones after the osteotomy; (b) fixation in the cylindrical mold; (c) specimen after casting with polyurethane resin FC 52; (d) preparation of tibia head with preinsertion of sensor attachment.

Figure 2 illustrates the four different steps undertaken to prepare each specimen prior to test. (a) Opening wedge proximal medial osteotomies were performed on each of the ten composite bones in the same way by an experienced surgeon, according to standard techniques of each angle-stable implant [6, 7]. (b) Then the composite tibias were cut, and only the proximal part of 300 mm length was further used by putting it in a cylindrical mold. All specimens were identically positioned with the help of a template and a centrical pinion at the bottom of the mold form. The inclination angle in the frontal and sagittal directions was chosen in a way that the tibia plateau was horizontal in both directions. The repeatability of the described positioning system was checked with different specimens and found to be less than 1 mm in all three dimensions. (c) The cylindrical pot was filled with a two-component polyurethane casting resin (FC 52). (d) After the hardening of the resin, the specimens were turned 180°, and the tibia heads with the osteotomy plates were positioned in shallow cylindrical molds. Two small thin metal plates were added in the molds to latterly attach the displacement sensors.

### 2.2. Mechanical Testing System

**Figure 3:**
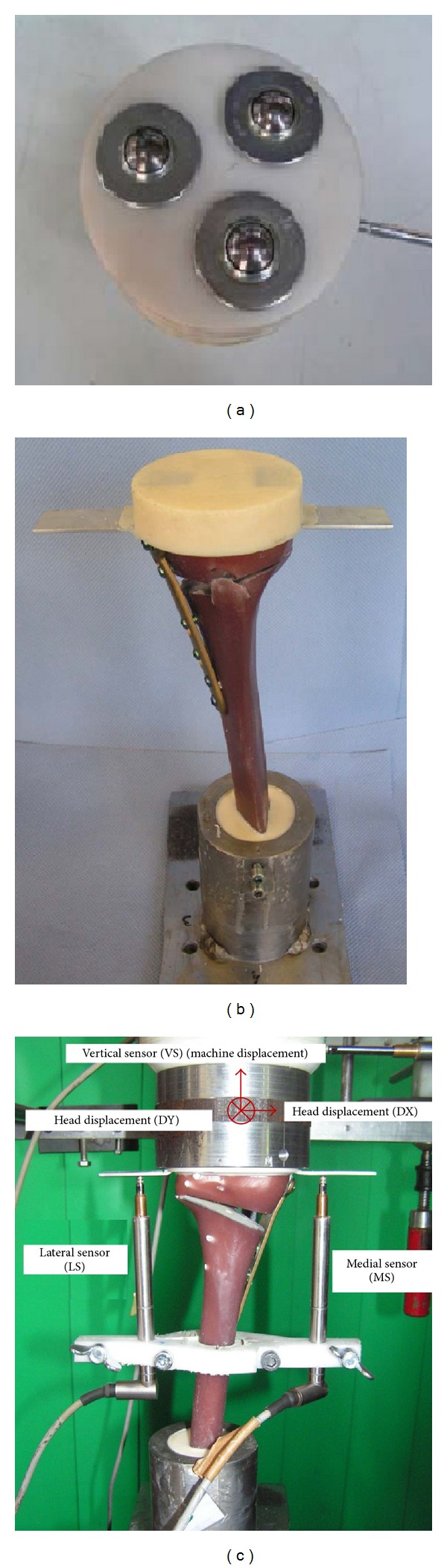
(a) Low friction sliding support to apply purely vertical forces. (b) Specimen before mounting to hydraulic press. (c) Specimen under test: the lateral and the medial sensors (LS and MS) register the relative displacement over 180 mm from the tibia head. The sensors DX and DY measured the horizontal displacement of the tibia head, while VS measured the vertical displacement.

The medial sensor MS measured the displacement in the frontal plane on the medial side of the tibia head and the second sensor LS at the lateral side. The purely vertical loading was applied to the tibia head through a freely movable support (Figure 3(a)), allowing any horizontal motion in the transversal plane using three freely rolling metal balls. The distal ends of the tibias were fixed to the vertical moving piston of the hydraulic testing machine. Two displacement sensors DX and DY were attached on the easily sliding support in order to measure the horizontal displacements of the tibial head in two perpendicular directions. A fifth displacement sensor VS measured the vertical displacement of piston of the testing machine (Figure 3(c)).

### 2.3. Testing Procedure

**Figure 4:**
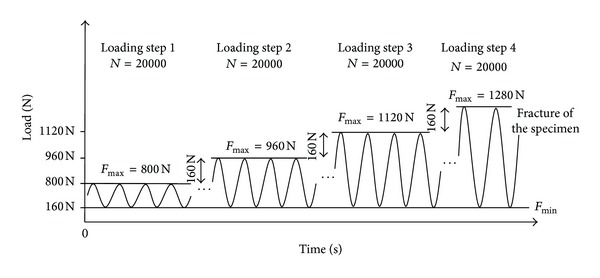
Scheme of the applied vertical sinusoidal force loading (load-controlled). After *N* = 20.000 cycles, the upper force is increased stepwise by 160 N until failure. The loading frequency was constant and set to 5 Hz.

Five specimens (Figure 3(b)) were prepared five Contour Lock HTO plates (group 1) and TomoFix plates (group 2). The groups were further subdivided depending on the type of test that should be performed (Table 1). Two specimens of each group were subjected to a static compression displacement-controlled (0.1 mm/s) single loading to failure, while the remaining three of each group were cyclically fatigue tested with compression sinusoidal loading. The force amplitude was kept constant with feedback control of the force signal within the hydraulic press. The displacement and force signals were measured and registered. Within the first load step, the forces varied sinusoidally from 160 N to 800 N. If no failure occurred after *N* = 20000 cycles, the upper force limit was increased by 160 N to proceed to the next step, while the lower limit was kept constant at 160 N. The testing frequency was fixed to 5 Hz (Figure 4). A similar testing protocol is standardized, for example, for hip joints [8–10], and was used by other authors for osteotomy plate testing, for example, by Agneskirchner et al. [11].

### 2.4. Type and Definition of Failure

A very important point is the definition of failure within these tests with purely compression forces, as the specimens are never completely destroyed. Pape et al. [12] used the following failure types which are summarized in Table 2. In case of linear systems, the displacement and force signals are varying sinusoidally with the same frequency but with different amplitudes and a phase shift. Hence if one plots force over displacement, an elliptical curve is obtained whose slope is proportional to the stiffness and whose enclosed area is proportional to the damping. If the specimen becomes unstable and starts to wobble, the width of this curve steeply increases. Hence the failure type 3 allows quantifying the wobble degree of the sample.

### 2.5. Static and Dynamic Loading

The contact forces in the knee joint during normal daily activities are nearly vertical. Of course the knee is also subject to horizontal forces and moments in the three anatomical planes (frontal, sagittal, and transversal planes), but the predominant loading direction is the vertical axis [13, 14]. Hence for the sake of simplicity, the comparative tests described herein were performed only by vertically loading the prepared specimens. Interesting to note that the vertical contact forces in the knee joint while slow walking are in average approximately 3 times the body weight [13], whereas under the foot it is 1 time once the body weight (BW). The muscles are contracting strongly thus allowing the stabilization of the knee-joint in order to transfer moments and nonvertical forces. This means for an individual of about 80 kg, the highest vertical compressive contact force will be approximately 2400 N during slow walking. Therefore and similar to the dynamic testing of hip joints, according to ISO 7206-4, -6, and -8 [8–10], a force controlled cyclic loading to failure pattern was defined and applied to three specimens of each group (Figure 4).

### 2.6. Mechanical Stiffness

**Figure 5:**
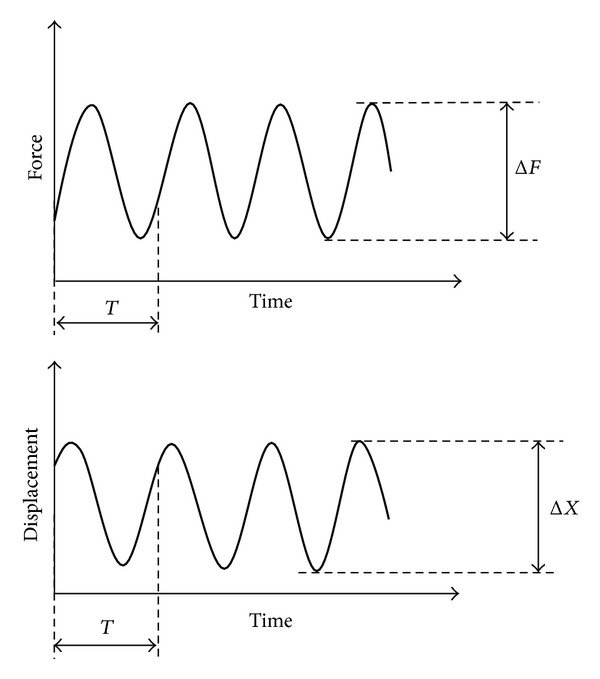
Definition of Δ*F* and Δ*X* to calculate the dynamic stiffness in the cyclic fatigue to failure tests.

During the cyclical loading, the evolution of the stiffness of the specimens was determined, as the mechanical stiffness is often used as damage indicator. In fatigue tests of complex structures, failure starts with local cracking, continues with crack growth, and ends up with collapse of parts of or the complete specimen. Hence the stiffness is frequently used as damage indicator and is here the ratio of peak to peak force Δ*F* = *F*_max⁡_ − *F*_min⁡_ to the measured peak to peak displacement Δ*X* in the same period *T* (Figure 5) as follows:

(1) $$\begin{array}{c} K=\frac{F_{\max}-F_{\min}}{\Delta X}. \end{array}$$

As there were multiple displacements measured (Figure 3(c)), for example, the lateral (LS) and the vertical displacements (VS) of the tibia head, two types of stiffness were defined: the lateral stiffness *K*_*L*_ and the vertical stiffness *K*_*v*_.

### 2.7. Plastic Deformation

**Figure 6:**
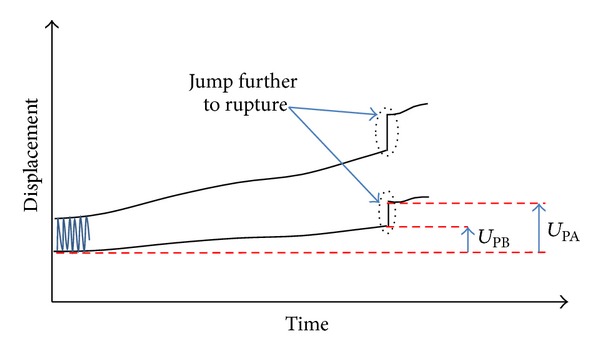
Definition of plastic deformation before and after failure: *U*_PB_ and *U*_PA_.

Any plastic deflection and the correlated plastic deflection angle would lead to a loss of correction and had to be checked for the failure type 1. The plastic deformation (Figure 6) was estimated here as the irrecoverable displacement with respect to the start of the tests at the minimal force of 160 N, considered as nearly zero force. Hence the plastic deformations could be measured online during the cyclic tests at any time, for example, before failure (*U*_PB_) and additionally after the gross failure, that is, in general the rupture of the lateral cortex (*U*_PA_).

The plastic deflection of the tibia plateau at a given time was defined as the resulting plastic displacements on the medial and the lateral sides in the frontal plane of the specimens.

**Figure 7:**
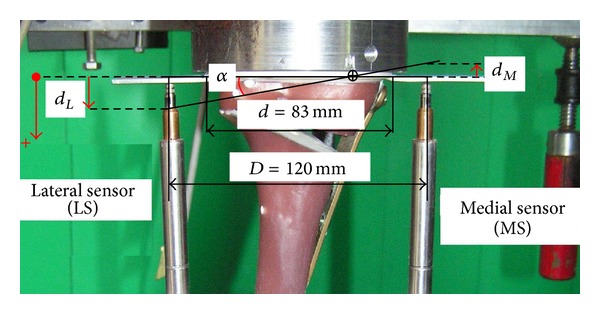
Definition of the positive displacement direction. In general the lateral displacement *d*_*L*_ was found to be positive with greater magnitude than the medial displacement *d*_*M*_ that was counted negative. Hence the deflection angle *α* was defined by means of the difference (*d*_*L*_ − *d*_*M*_).

According to Figure 3(c), two sensors LS and MS register the lateral displacement *d*_*L*_ and the medial displacement *d*_*M*_, respectively. Though the test force was strictly vertical, the specimens deformed not purely vertical, resulting in unequal displacement values for *d*_*L*_ and *d*_*M*_ (Figure 7). An deflection angle (in radians) was defined and could be calculated at any time as

(2) $$\begin{array}{c} \alpha =\frac{\left|d_{L}-d_{M}\right|}{D}. \end{array}$$

According to the definitions of plastic deformations and the failure type 1, a loss of correction occurs when

(3) $$\begin{array}{c} \frac{d}{D}\left|d_{Lp}-d_{Mp}\right|>2\;\text{mm}; \end{array}$$

that is, if *α*_*p*_ > 0.024 rad or 1.4°, with the index “*p*” meaning plastic.

## 3. Results and Discussion

### 3.1. Static Loading to Failure

**Figure 8:**
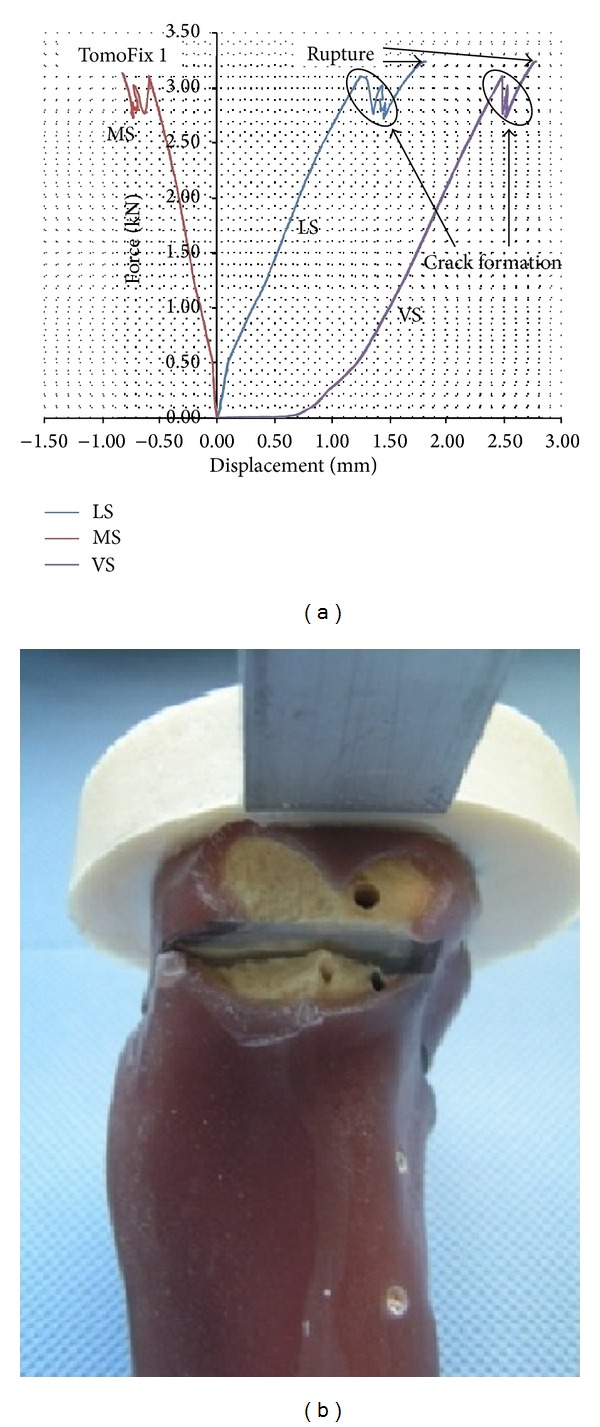
(a) Lateral (LS), medial (MS), and vertical displacements of the tibia head during the static load to failure tests. (b) Picture of the contralateral cortex after rupture that was generally preceded by the occurrence of cracks (case of TomoFix1).

During the static loading to failure tests, all specimens failed due to a fracture of the contralateral cortical bone (failure type 2). In general crack formation was observed before collapse (Figure 8). The medial displacement (MS) is negative while the lateral displacement (LS) is positive, and the magnitude of the lateral displacement is approximately the double of the medial displacement. Hence the tibial plateau of all the specimens rotated (Figure 7).

Table 3 gives the summary of the recorded forces, the corresponding lateral displacements, the deflection angle, and the failure types observed. A sudden fracture without cracking before, but a rotation of the tibia head, was observed in the case of the Contour Lock 2 specimen.

### 3.2. Fatigue Loading to Failure Tests

**Figure 9:**
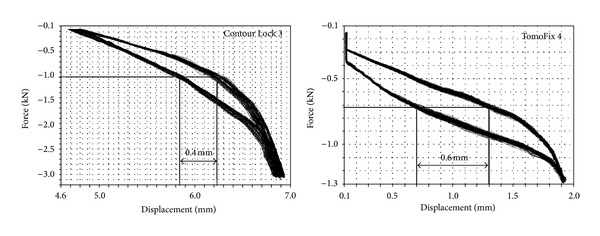
Typical hysteresis loops after rupture of the contralateral cortex (force versus lateral displacement) showing the occurrence of failure type 3 in the case of the TomoFix 4 (load step 3 or shorter LS3). Contour Lock 3 did not show this failure type even in load step 14 (LS14).

The maximum reached load step and the number of cycles reached before the collapse of the specimens were recorded. As for the static analysis, all the specimen failed in the fatigue tests due to a rupture of the contralateral cortical bone. No screw crack was observed. The relation between the applied loads and the resulting lateral displacements was plotted in order to check failures of type 3 (Table 2) after the rupture of the lateral cortex. The lateral displacement (LS) was used, because it was higher than the medial displacement (MS), and because the rupture occurred always on this side.  Figure 9 shows typical hysteresis loops obtained by plotting the applied force versus the lateral displacement in cases of the specimens TomoFix 4 and the Contour Lock 3. A failure type 3 can be observed for TomoFix 4.

Table 4 gives the summary of the types of failure observed, the maximal forces, the number of cycles at the moment of failure, and the corresponding vertical and lateral stiffnesses before failure. The mean values of the maximal loads were 1440 N and 2190 N for the TomoFix and the Contour Lock groups, respectively. The vertical and the lateral stiffnesses were in average, respectively, 2000 N/mm and 1930 N/mm for the TomoFix plate-fixated specimens versus 2367 N/mm and 3133 N/mm for the Contour Lock group. The number of load cycles applied to the specimens of the TomoFix group until complete rupture of the lateral cortex were in average 8000 cycles corresponding to the load step 5 (LS5) versus 160000 cycles and the load step 4 (LS4) for the Contour Lock plate-fixated specimens.

**Figure 10:**
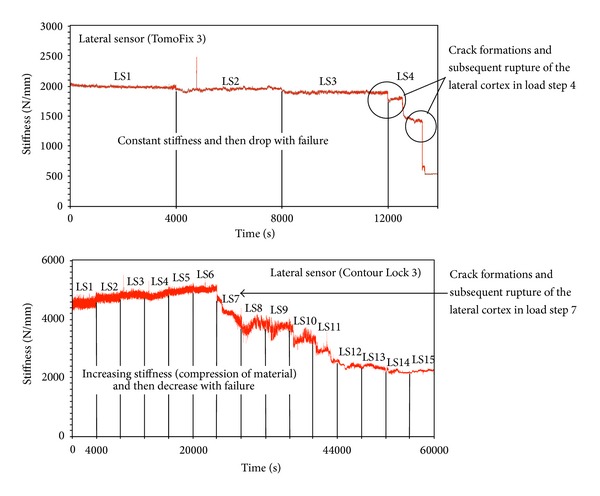
Typical evolution of the lateral stiffness for a TomoFix and Contour Lock during fatigue to failure tests.

Figure 10 shows two typical evolutions of the stiffness until failure of the specimen. With the crack formation, the stiffness drops and then drops again with the final failure.

**Figure 11:**
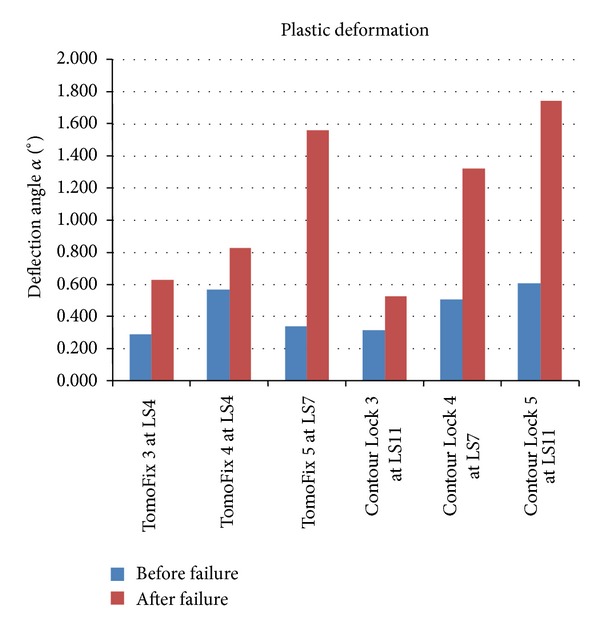
: Plastic deformation angle (loss of correction) for all specimens during the fatigue failure tests before and after failure. As stated above; 1.4° (or 2 mm) is generally considered as maximum acceptable limit. (LSn) indicates that the failure occurred at the load step n.

The determined plastic deflection's angles before and after the rupture of the lateral cortex are summarized in the graph of Figure 11. The load history according to Figure 4 is indicated with the Load Step number (LSn) at which the failure occurred.

## 4. Discussion

The aim of this study was to compare the static and fatigue strength provided by the Contour Lock HTO plate and the TomoFix plate, both designed for medial opening wedge high tibial osteotomy. Ten fourth-generation tibial bone composites (Sawbones) underwent a medial opening wedge HTO, for which five TomoFix Tibial Head Plates and five Contour Lock HTO Plates were used. Static and dynamic tests were performed on the resulting bone-implant constructs. The key finding of this is that the new introduced Contour Lock plates offer a higher stability in case of maximum dynamic loading of the bone-implant constructs. However, load values at the time of construct failure were above physiologic conditions in the case of static loading.

The opposite cortex failed in all cases, very often quickly after the first important cracking. Other biomechanical comparative studies mentioned the fracture of the lateral cortical bone as decisive failure [11, 15–17]. No visible cracks of more than 1 mm of the screws (failure type 3) were observed. The displacement at the medial side was always smaller than the lateral displacement. This difference was observed in all bone-implant constructs regardless of the performed test. Furthermore, no failure of the implants was detected. Both plates are thus sufficiently resistant.

In the static compressive tests, the TomoFix plate failed in average at 3400 N and the Contour Lock plate at 3550 N. The difference is small, and those mean values are bigger than the tibiofemoral contact force while slow walking, as this force is about 3 times the body weight [13, 14], that is, 2400 N for an individual weighing 80 kg. However, the Contour Lock showed higher displacement and deflection angle values at the maximum static force, suggesting that the TomoFix seems a bit better suited for these static tests. But the static tests do not take into account the eventual effects of fatigue, which take place when the bone-implant constructs are repetitively loaded.

The main goal of the cyclic testing was to simulate repetitive loads to the tibia head during daily activities and thus analyze the stability of the osteotomy plates. Although the failure mode observed during cyclic loading was identical to that observed during the static test, the maximum forces at the moment of failure (in average 2190 N for the specimens with the Contour Lock plates and 1440 N with the TomoFix plates) were significantly lower than those measured in the static case. For specimens of the Contour Lock group, the failure occurred after 160000 cycles in average versus 80000 cycles for the specimens of the TomoFix group. This shows that the performed osteotomy would fail by fatigue after a small number of full-charged cycles, if there was no healing process. All these failure loads are far less than the average axial force of about 2400 N in the knee during normal walking for an individual weighing 80 kg. This implies that full dynamical loading directly after the osteotomy cannot be recommended. The specimens with TomoFix plates were significantly weaker than those with the Contour Lock plates regarding these parameters. However one should note that the TomoFix used is designed for small adults weighing up to 65 kg, and the Contour Lock is not restricted.

The stiffness was proposed as a damage indicator here and it typically showed first a slight increase due to the compacting of the composite material and a more or less pronounced decrease after rupture of the contralateral cortex. The increase of the stiffness before the rupture was more pronounced for bone-implant constructs of the Contour Lock group. By the TomoFix specimens, the stiffness was first nearly constant and then decreased with the occurrence of the rupture. Stoffel et al. [16] concluded that stability is dependent largely on an undamaged lateral hinge of the constructs. This statement is in agreement with the above mentioned behavior of the bone-implant constructs regarding the stiffness in this study.

The failure type 3 (maximal displacement within a hysteresis greater than 0.5 mm during the dynamic testing) intends to limit the tolerable wobble degree of the bone-implant construct under dynamic loading. This type of failure appeared twice within the TomoFix group and once within the Contour Lock group thus providing the better stability with respect to this parameter. This is due to the fact that the Contour Lock plate is wider and thus anchored at a bigger surface (more dispersed separation of its screws) of the tibia head than the TomoFix plate.

As a positive outcome of the osteotomy depends closely on the maintenance of the primary correction angle, it is important to estimate the plastic deformation during a dynamic loading of the bone-implant constructs, thus determining the loss of correction, which corresponds to irreversible deformation of the specimen. Plastic deformation angles, leading to the loss of correction during dynamic testing, were observed in the case of the TomoFix 5 and of the Contour Lock 5, respectively, at load steps 7 and 11. This means in this case that the Contour Lock better conserves the correction than the TomoFix. However this conclusion is valid if there was no bone healing prior to the fatigue rupture.

Agneskirchner et al. [11] performed single load to failure and cyclical load to failure tests to compare the stability of four different implants; three spacer plates were shorter than the fourth medial plate fixator, which was a TomoFix plate. The displacements at the osteotomy gap were smaller for the specimens with the TomoFix for all performed tests. This concords with the results obtained in our study regarding the static test. Agneskirchner et al. [11] reported the better stability for the TomoFix and concluded that short designed implants are inferior to longer designed plates. However none of the short spacer plates was a T-shaped plate with a wider proximal end like the Contour Lock plate of the present study. A study by Stoffel et al. [16] that compared the TomoFix to the Puddu plate (rectangular short spacer plate) reported also a better axial stability for the TomoFix. But in our study the new Contour Lock showed superior behavior in the cyclic testing, which is considered more important than the static loading tests.

While considering the findings of this study, one should also take into account the small number of specimen used during this study. Of course one may discuss the specimen's preparation and the used testing procedure compared to the reality in the human knee, where muscle forces, small bending moment, and friction forces are present and may influence the results. However this reality is difficult to simulate in a testing machine, and therefore caution is recommended. A possible solution might be to simulate the muscle forces with appropriate multi-body-system software and apply them to a finite element model of the tibia head with the osteotomy plate. Then the stresses of the simulated real situation and of the test situation could be compared permitting an assessment of the testing setup.

As the Contour lock plates are wider, the better stability they offered regarding the cyclic test is probably due to the larger distance between their fixation screws and their wider T-shaped proximal ends. One may also relate the low degree of stability of the TomoFix small stature plate compared to the Contour Lock to its small geometry. It would be therefore interesting to perform another comparative study with the TomoFix standard plate, which has no weight restriction and is longer than the small stature plate.

## 5. Conclusion

In summary, the Contour Lock plate provided higher stability than the TomoFix small stature plates for the medial opening wedge HTO regarding the dynamic loading of the bone-implant constructs in terms of occurrence of the dominant failure, that is, the rupture of the lateral cortex. Both plates are able to maintain the osteotomy for a static load up to 3000 N, which is normally more than the maximal axial force in the knee during walking. Both plates provide sufficient strength, because the lateral cortex is weaker than the plate. It is hence decisive for the mechanical strength in the fatigue to failure tests how the tibia head is fixed to and guided by the plate. In this sense the Contour Lock plate showed better behavior than the TomoFix small stature plate.

## Figures and Tables

**Table 1:** Specimen assignment, depending on the performed test.

Performed test	Group 1; *n* = 5 specimens	Group 2; *n* = 5 specimens
Static: single loading to failure test	Contour lock 1	TomoFix 1
	Contour lock 2	TomoFix 2

Dynamic: cyclical fatigue failure test	Contour lock 3	TomoFix 3
	Contour lock 4	TomoFix 4
	Contour lock 5	TomoFix 5

**Table 2:** Used failure types and their defining criteria.

Failure type	Criteria
1	Visible yielding and dislocation/tilt of the tibia plateau of more than 2 mm. This criterion can only be checked in the unloaded condition and addresses the unwanted loss of correction after surgery.

2	Visible collapse of lateral cortex. Small hairline cracks are not considered as failure.

3	Maximal displacement range of more than 0.5 mm within one hysteresis loop in the case of cyclic testing only.

4	Cracks of the screws of more than 1 mm.

**Table 3:** Comparison of the four specimens subjected to static tests at the moment of the first cracking and the ultimate loading state. No cracks were detected prior to collapse in the case of the Contour Lock 2 specimen.

Specimen	Crack load/ultimate load [kN]	Medial displ. at crack and ultimate loads [mm]	Lateral displ. at crack and ultimate loads [mm]	Deflection angle at crack and ultimate loads (°)	Failure type
TomoFix 1	3.1/3.2	0.60/0.86	1.25/1.82	0.88/1.28	2
TomoFix 2	3.2/3.6	0.43/0.60	1.55/2.25	0.94/1.36	2
Contour Lock 1	2.4/3.2	0.60/0.52	2.45/3.85	1.46/2.09	1 and 2
Contour Lock 2	—/3.9	—/0.50	—/4.15	—/2.22	1 and 2

**Table 4:** Summary of fatigue failure tests (all values before failure): max. load, vertical and lateral stiffness and number of cycles. The Contour Lock plate shows better performance in all investigated parameters.

Specimen	Maximal load [N]	Vertical Stiffness *K*_*V*_ [N/mm]	Lateral Stiffness *K*_*L*_ [N/mm]	Number of cycles	Failure type
TomoFix 3	1280	2200	2000	>60000	2, 3
TomoFix 4	1280	1750	1500	>60000	2, 3
TomoFix 5	1760	2000	2300	>120000	1, 2
Contour Lock 3	2400	2100	4400	>200000	2
Contour Lock 4	1760	2300	2400	>120000	2
Contour Lock 5	2400	2700	2600	>200000	1, 2, 3
